# Understanding the role of entropy in designing high-performance thermoelectrics

**DOI:** 10.1126/sciadv.aed6943

**Published:** 2026-07-03

**Authors:** Subrata Ghosh, Soongyu Kwon, Wenjie Li, Dipika Mandal, Amin Nozariasbmarz, Erik Furton, Marcia Ahn, Sumanta K. Karan, Allison M. Beese, Yi Xia, Shashank Priya, Bed Poudel

**Affiliations:** ^1^ Department of Materials Science and Engineering, Pennsylvania State University, University Park, PA 16802, USA.; ^2^ Department of Mechanical and Materials Engineering, Portland State University, Portland, OR 97201, USA.; ^3^ Department of Biochemistry, Chemistry & Physics, Georgia Southern University, Statesboro, GA 30458, USA.; ^4^ Center for Advanced Materials Science, Georgia Southern University, Statesboro, GA 30458, USA.; ^5^ Department of Mechanical Engineering, Rowan University, Glassboro, NJ 08028, USA.; ^6^ Department of Chemical Engineering and Materials Science, University of Minnesota, Minneapolis, MN 55455, USA.

## Abstract

The design of high-entropy alloys is effective in lowering thermal conductivity but often reduces carrier mobility, thereby limiting electrical transport properties. Understanding the relationship among configurational entropy, thermal conductivity, and carrier mobility in high-entropy alloys is critical for enhancing thermoelectric performance. We demonstrate that a systematic and substantial increase in configurational entropy in half-Heusler alloys leads to an asymptotic reduction of lattice thermal conductivity. The reduced phonon group velocity and enhanced phonon scattering induced by atomic-scale chemical disorder result in a low lattice thermal conductivity of 2 watts per meter per kelvin at room temperature, with an achievable minimum of about 1.48 watts per meter per kelvin as disorder further increases, approaching the amorphous limit. Single-phase stabilization, along with optimized carrier mobility, is essential to preserve high electrical conductivity. Our results provide fundamental insights into integrating specific strategies with high-entropy design to simultaneously achieve low thermal conductivity and high thermoelectric performance, advantageous for high-temperature thermoelectric power generation applications.

## Teaser

Entropy design enhances thermoelectric performance.

## INTRODUCTION

Solid-state thermoelectric (TE) generators (TEGs) can harvest electricity from the waste heat and decrease the utilization of fossil fuels through waste-heat power generation (*1*–*6*). The performance of a TE material relies on the dimensionless figure of merit $\left(\mathrm{zT}\right)=\frac{S^{2}\sigma }{\kappa }T$, where *S*, σ, κ, and *T* represent the Seebeck coefficient, the electrical conductivity, the thermal conductivity, and absolute temperature of the material, respectively (*4*, *5*, *7*). Here, κ is the sum of the electronic (κ_e_) and lattice part (κ_L_) of the total thermal conductivity. A high *zT* in a TE material is the result of a high power-factor ($\mathrm{PF}=S^{2}\sigma$) and/or low κ. However, the strong intercorrelation of TE parameters (*S*, σ, and κ) challenges the tuning of *zT* in a given material (*4*, *8*). To decouple TE parameters, various strategies such as nanostructuring (*7*, *9*), band engineering (*10*, *11*), grain boundary manipulation (*12*, *13*), defect engineering (*14*, *15*), and resonant dopant (*16*) have been used in aiming to increase *S* and/or σ or to decrease κ. However, for the high-temperature power-generation applications, TE materials with simultaneously high *PF* and *zT*, and low κ are attractive. This combination of properties is not typically found among potential high-temperature TE material systems, including half-Heusler (hH) alloys.

High-entropy engineering (*17*–*21*), a recent successful strategy to optimize the electrical and thermal transport properties, can drive intrinsically low κ_L_ in the TE materials (*22*). However, it is crucial to understand the trend of κ_L_ with increasing configurational entropy (Δ*S*_config_) of a material system. To maximize the *zT*, it is essential to preserve the carrier mobility (μ) to maintain high σ, which is often compromised by a highly disordered crystal structure. Therefore, understanding the role of high-entropy engineering and establishing its relationship with κ_L_ and μ is critical for developing advanced TE materials with low κ_L_ while maintaining excellent electrical transport properties. The key aspects of entropy engineering in a material are as follows (*23*, *24*): (i) The incorporation of multiple elements at a particular Wykoff site enhances the mixing entropy of the alloy, which reduces the Gibbs free energy and stabilizes the single-phase, and (ii) the presence of multiple elements induces severe lattice distortion from chemical fluctuation, increasing the high-frequency phonon scattering. Simultaneously, single-phase stabilization weakens the phase-boundary electron scattering. Nevertheless, high-entropy alloys (Δ*S*_config_ ~1.5 R or higher), in conjunction with their unique feature of “single-phase crystal and highly distorted lattice,” show extraordinary functional and structural properties compared to conventional alloys (*25*). This provides an opportunity to tailor specific properties such that there is an optimum response for a given set of boundary conditions.

Conventional hH alloys are popular as potential high-temperature TE materials due to their exceptionally high *PF* and superior thermal and mechanical stabilities (*3*, *26*–*31*). Although hH materials demonstrate high *zT*, their high κ hinders further optimization of *zT*. This also makes it particularly infeasible to generate a large temperature gradient (Δ*T*) across the fabricated TEGs to maximize the waste heat to electricity conversion efficiency (η). In our recent study, we demonstrated that a high-entropy design strategy on a p-type hH material, MFeSb (M = Nb, Ta, Ti, and V in equimolar), shows a high zT with a large *PF* and low κ (*20*). Inspired by this result, we sought to explore the trend of κ_L_ with increasing Δ*S*_config_, approaching the limiting value of κ_L,_ and to investigate the potential impact of increasing Δ*S*_config_ on carrier mobility of the material.

**Fig. 1.**
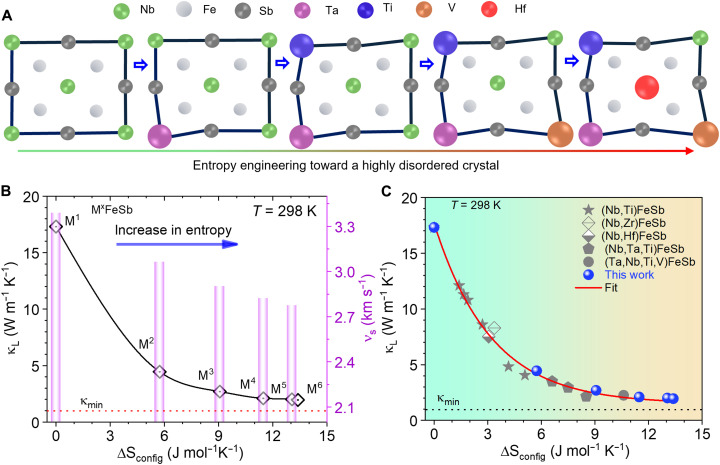
High-entropy engineering and low lattice thermal conductivity. (**A**) A schematic representation of a substantial increase in Δ*S*_config_ leads to a highly disordered crystal. (**B**) κ_L_ of M^x^FeSb alloys at room temperature (~298 K) and their corresponding sound velocity (ν_s_) as a function of Δ*S*_config_. (**C**) Room temperature κ_L_ of M^x^FeSb and various p-type (Nb, Ta)FeSb hH TE materials (*28*, *31*, *35*, *36*) are plotted with the associated Δ*S*_config_. The κ_L_ versus Δ*S*_config_ is fitted with the equation, $\kappa_{L}=\kappa_{L0}+bc^{∆S_{\text{config}}}$ where κ_L0_ is the achievable minimum κ_L_ in the high-entropy limit. The theoretical minimum thermal conductivity (κ_min_) is calculated using the Cahill model (*33*, *34*), as shown by a dashed line (details are in the Supplementary Materials, note S2).

In the present study, we chose NbFeSb, an hH semiconductor with an indirect bandgap of 0.53 eV (*32*), as a case study to explore the role of entropy in optimizing TE performance through balancing thermal conductivity and mobility of the material. We demonstrate that a systematic design of high-entropy–driven M^x^FeSb hH with Nb, Ta, Ti, V, and Hf atoms on M^x^ site via increasing Δ*S*_config_ (Fig. 1A) (where M^1^: Nb, M^2^: Nb_0.5_Ta_0.5_, M^3^: Nb_0.33_Ta_0.33_Ti_0.33_, M^4^: Nb_0.25_Ta_0.25_Ti_0.25_V_0.25_, M^5^: Hf_0.1_Nb_0.225_Ta_0.225_Ti_0.225_V_0.225_, and M^6^: Hf_0.2_Nb_0.2_Ta_0.2_Ti_0.2_V_0.2_), can simultaneously reduce the κ and sustain the high σ through the single-phase stabilization along with the optimization of *n* and μ. We find that as the Δ*S*_config_ increases, the κ_L_ decreases drastically and approaches the theoretical minima (*33*, *34*) due to the reduction of phonon group velocity (*v_g_*) (Fig. 1B). The κ_L_ of M^x^FeSb demonstrates a similar trend with Δ*S*_config_, even when the κ_L_ of various state-of-the-art MFeSb-based hH TE materials are considered (Fig. 1C). This suggests that the reduction of κ_L_ with Δ*S*_config_ is asymptotic and further reduction is unlikely. However, the increase in Δ*S*_config_ shows a compromised μ compared to the low-entropy TE systems. From these results, it can be found that through simultaneous optimization of Δ*S*_config_ and μ, M^4^FeSb with a large Δ*S*_config_ ~1.4 R demonstrates both exceptionally low κ_L_ (~2 W m^−1^ K^−1^ at 300 K) and a high *zT* (~1.25 at 873 K), which outperforms most of the conventional hH materials (*28*, *31*, *35*, *36*). Therefore, our results provide guidance incorporating other conventional approaches with the appropriate entropy consideration to tailor the *zT* of various kinds of TE materials.

## RESULTS

**Fig. 2.**
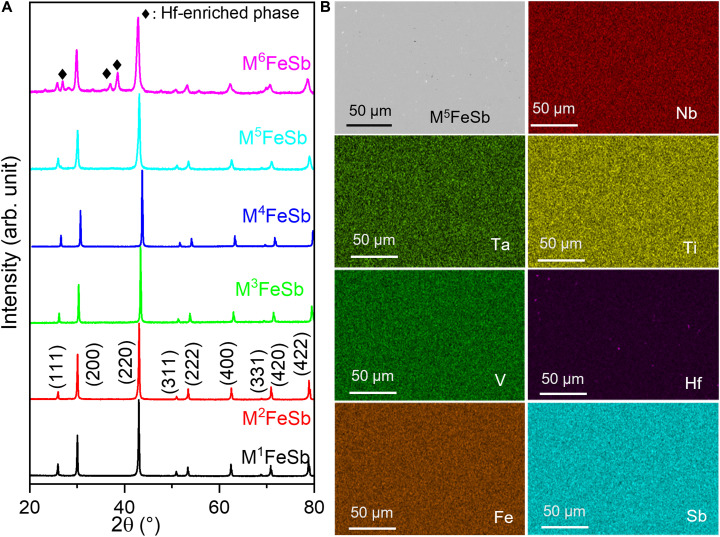
Crystal structure and microstructure analysis. (**A**) XRD pattern at room temperature shows that the samples M^1^FeSb to M^5^FeSb have a single hH phase. For M^6^FeSb, when the Hf concentration is high, a secondary trace of Hf-enriched phase has evolved. (**B**) FESEM image and the EDS mapping show the homogenous distribution of constituent elements for M^5^FeSb alloy within a micrometer length scale.

hH materials have a general formula XYZ, where X atoms fill the octahedral voids at (0, 0, and 0) around the tetrahedral framework formed by Y atoms at (1/4, 1/4, and 1/4) and Z atoms at (1/2, 1/2, 1/2) in a Zintl chemistry principle (*4*). This enables the design of high-entropy hH alloys with diverse elemental compositions, particularly, favoring single-phase formation when the elements are introduced in the X site. Through alloying of Nb, Ta, Ti, V, and Hf on the X site, we have synthesized a series of NbFeSb-based hH alloys (M^x^FeSb) (see Materials and Methods). M^5^FeSb and M^6^FeSb have the Δ*S*_config_ >1.6 R in a single Wyckoff site, which belong to the high-entropy regime. The x-ray diffraction (XRD) patterns at room temperature (~298 K) show that M^1^FeSb to M^5^FeSb demonstrate single-phase stabilized hH phase (F$\bar{4}$3 m, cubic structure), and while a high percentage of Hf content is introduced in the system (M^6^FeSb), a secondary trace of Hf-enriched phase is developed (Fig. 2A).

To obtain further insight into the crystal phase, we performed microstructural analysis using field-emission scanning electron microscopy (FESEM). The area mapping by the energy-dispersive x-ray spectroscopy (EDS) indicates the homogenous distribution of constituent elements for the high-entropy M^5^FeSb alloy on a micrometer length scale (Fig. 2B). Further insight into the microstructure reveals the Hf-rich nanoprecipitates in the material. However, nanoprecipitates are usually a common feature in most of the functional high-entropy materials and are beneficial to induce midfrequency phonon scattering to reduce κ_L_. The chemical composition revealed by EDS as Nb_0.225_Ta_0.23_Ti_0.224_V_0.225_Hf_0.105_Fe_0.99_Sb_1_ is very close to the nominal composition of the material. EDS mapping for other single-phase compositions, M^1^FeSb to M^4^FeSb, demonstrates the homogeneous distribution of elements (figs. S1 to S4). In contrast, we have observed a secondary Hf-rich phase in high-entropy M^6^FeSb as evident from EDS mapping (fig. S5). It is expected that the phase boundary at the micrometer length scale can help induce acoustic phonon scattering, but electrical transport is most likely affected by the additional electrons scattering across the phase boundary. The electron backscatter diffraction (EBSD) for all the single-phase samples (fig. S6) reveals that the high-entropy effect substantially reduces the average grain sizes from ~700 nm in M^1^FeSb to ~100 nm for M^4^FeSb. However, the grain size increases to ~400 nm in the M^5^FeSb sample when the Hf is introduced into the system. The incorporation of Hf into the high-entropy system has previously been reported to promote grain growth, consistent with our observation (*37*).

**Fig. 3.**
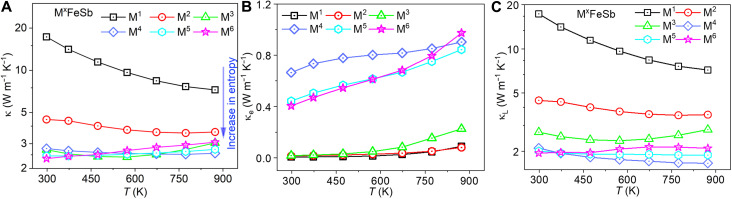
Thermal transport properties of M^x^FeSb alloys. Temperature dependence of (**A**) κ*,* (**B**) κ_e_, and (**C**) κ_L_ of entropy-engineered M^x^FeSb alloys.

High-entropy engineering typically leads to a notable increase in phonon scattering due to the enhanced disorder arising from chemical fluctuations of the constituent elements, which leads to intrinsically low κ_L_ in the high-entropy–engineered TE materials (*22*). Entropy engineering to reduce κ_L_ is highly effective in this hH system and can guide the design of low κ_L_ hH materials (Fig. 1). The total κ at room temperature reduces from 17.31 to 2.45 Wm^−1^ K^−1^ as the composition evolves from low-entropy M^1^FeSb to high-entropy M^5^FeSb (Fig. 3A), clearly demonstrating a reduction of κ with increasing Δ*S*_config_. For the phase-separated high-entropy composition, M^6^FeSb, κ further reduces to 2.34 Wm^−1^ K^−1^. The reduction of κ with increasing Δ*S*_config_ is attributed to a decrease in the lattice part of κ. κ_L_ is evaluated by subtracting κ_e_ from the total κ, where κ_e_ (Fig. 3B) is estimated using the Wiedemann-Franz relationship, κ_e_ = Lσ*T*. L is the Lorenz number evaluated using the single parabolic band approximation (fig. S7) (*38*). As shown in Fig. 3C, κ_L_ is substantially suppressed with increasing Δ*S*_config_ of the material. At room temperature, κ_L_ for M^1^FeSb is about 17.3 Wm^−1^ K^−1^, which is reduced to about 2 Wm^−1^ K^−1^ for M^5^FeSb, achieving a reduction of 88.4%. This effect of high-entropy engineering in the reduced κ_L_ is further illustrated by calculating the disorder parameter, Г (fig. S8), which arises from the large differences in atomic mass and radius among the constituent elements; the observed enhanced value of Г with Δ*S*_config_ suggests stronger defect-scattering of phonons (*28*), thereby suppressing κ_L_. Moreover, on the basis of kinetic theory, κ_L_ of bulk materials can be expressed as $\kappa_{L}=\frac{1}{3}C_{v}v_{g}l=\frac{1}{3}C_{v}v_{g}^{2}\tau$, where *C*_*v*_ is the specific heat capacity, *v*_*g*_ is the phonon group velocity, *l* is the phonon mean free path, and τ is the phonon relaxation time (*22*, *27*). Extensive studies have demonstrated that the contraction of *l* and diminishing τ can reduce κ_L_ (*39*). Likewise, the low $v_{g}$ indicates intrinsically low κ_L_ due to lattice softening (*40*), which can be approximated by the low-frequency sound velocity (ν_s_) of the material. Hence, we measured the average sound velocity (*v*_s_) of the materials, which decreases monotonically with increasing Δ*S*_config_ (Fig. 1B). This reveals that high-entropy engineering reduces *v*_*g*_, leading to suppressed κ_L_ in M^x^FeSb. In addition, we also observed a substantial reduction in grain size as the composition evolved from M^1^FeSb to M^4^FeSb. The reduced grain size can be attributed to the high-entropy effect and increase in chemical complexities. Smaller grains introduce additional grain boundary scattering, enhancing phonon scattering, particularly midfrequency phonons, and thereby contribute to suppressed κ_L_. Room-temperature κ_L_ values of M^x^FeSb together with those of reported low-to-mid entropy MFeSb systems (*28*, *31*, *35*, *36*) are plotted with Δ*S*_config_, which shows the reduction of κ_L_ with Δ*S*_config_ is asymptotic in nature (Fig. 1C). We have fitted κ_L_ versus Δ*S*_config_ using the empirical asymptotic relation, $\kappa_{L}=\kappa_{L0}+bc^{∆S_{\text{config}}}$ where κ_L0_ is the achievable minimum value of κ_L_ in the high-entropy limit. *b* and *c* are fitting parameters with 0 < c < 1, ensuring the asymptotic reduction. From the fit, we have extracted the minimum κ_L_ of the MFeSb system that can be achieved via entropy engineering, which is estimated to be ~1.48 ± 0.3 W m^−1^ K^−1^ at room temperature.

**Fig. 4.**
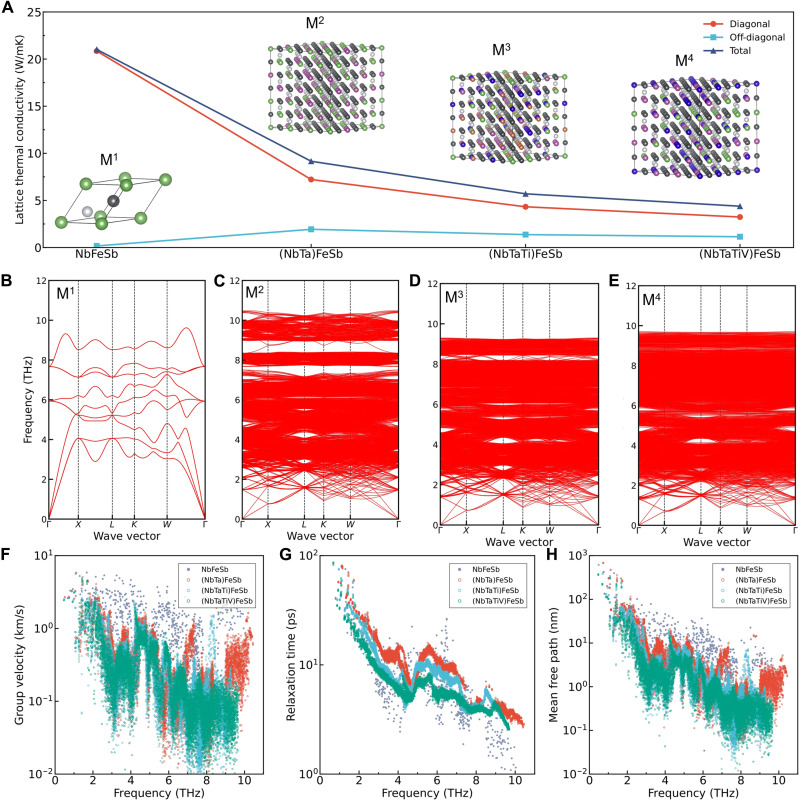
Calculated lattice thermal conductivity of M^x^FeSb. (**A**) Calculated κ_L_ at 300 K for M^1^FeSb, M^2^FeSb, M^3^FeSb, and M^4^FeSb, including diagonal, off-diagonal, and total components based on the unified theory of thermal transport in crystals and glasses (*41*). Corresponding crystal structures are also displayed. The calculated phonon dispersions for (**B**) M^1^FeSb, (**C**) M^2^FeSb, (**D**) M^3^FeSb, and (**E**) M^4^FeSb. The mode-resolved (**F**) group velocity, (**G**) relaxation time, and (**H**) mean free path for all four compounds.

To understand the lattice thermal transport behavior across the studied high-entropy systems, we show in Fig. 4A the calculated κ_L_ of M^1^FeSb, M^2^FeSb, M^3^FeSb, and M^4^FeSb at 300 K using the unified theory of thermal transport in crystals and glasses from first principles, effectively accounting for both propagating phonons (diagonal terms) and localized diffusons (off-diagonal terms) (*41*). The insets in Fig. 4A show the corresponding atomic structures used in the simulation. Specifically, low-entropy NbFeSb is an ordered structure that contains three atoms in its primitive cell, whereas the higher-entropy compounds M^2^FeSb, M^3^FeSb, and M^4^FeSb are disordered, and each consists of 324 atoms in their conventional cells. For the disordered structure, we generated atomic occupations by means of the special quasirandom structure approach (*42*) to ensure the configurational disorder. The choice of supercell size is based on our test to converge the calculated κ_L_ for disordered structures. Specifically, we tested cell sizes of 48 and 96 atoms for M^2^FeSb and M^4^FeSb, and 72 and 108 atoms for M^3^FeSb. With increasing cell size, we observed a reduction in the total κ_L_, including both diagonal and off-diagonal components (*41*). To obtain more converged results, we increased the number of atoms up to 324 for all high-entropy compounds, which was the maximum feasible size due to computational limitations. With these finalized supercells, we analyzed how κ_L_ evolves with increasing configurational complexity. As shown in Fig. 4A, we see that as the number of constituent elements increases, the total κ_L_ decreases and tends to flatten. The difference in conductivity between NbFeSb and the higher-entropy compounds is substantial; however, the reduction becomes less pronounced with the addition of more elements. For example, the difference in κ_L_ between M^3^FeSb and M^4^FeSb is much smaller than that between M^1^FeSb and M^2^FeSb. We also notice that while the off-diagonal component is enhanced in all higher-entropy compounds compared to M^1^FeSb, it does not increase monotonically with the number of constituent elements, indicating its crucial dependence on the chemical composition. This behavior can be attributed to the increased complexity of the phonon spectra in the high-entropy compounds. As shown in the phonon band structures (Fig. 4, B to E), M^2^FeSb, M^3^FeSb, and M^4^FeSb exhibit a substantially larger number of phonon branches with denser and more overlapped dispersions due to the increased number of atoms in the unit cell, which provides additional phonon hopping channels and enhances the off-diagonal contribution to κ_L_. However, even when the off-diagonal component increases, the total κ_L_ continues to decrease and flatten. This trend suggests that increasing structural complexity effectively suppresses κ_L_ via reducing the contributions from the propagating phonons but with diminishing returns beyond a certain level of disorder.

To investigate the origin of these trends, we computed the phonon dispersion relations of all four compounds, shown in Fig. 4 (B to E). Compared to M^1^FeSb, the acoustic phonon branches of the high-entropy compounds are substantially suppressed, as evidenced by a substantial reduction in their frequency range. All four materials show intersections between acoustic and transverse optical (TO) modes. However, in the higher-entropy compounds, M^2^FeSb, M^3^FeSb, and M^4^FeSb (Fig. 4, C to E), the TO modes are positioned closer to the transverse acoustic modes than in M^1^FeSb (Fig. 4B). Such an overlap between optical and acoustic modes is a signature for increased scattering of acoustic modes, contributing to reduced κ_L_ (*43*). Concerning the highest vibrational frequency, we find that the introduction of Ta into NbFeSb increases the highest phonon frequency. In contrast, adding Ti lowers this maximum frequency, while the subsequent addition of V raises it slightly again, although not to the level observed in M^2^FeSb. This trend indicates that the highest phonon frequencies are sensitive to the specific chemical elements present and do not follow a monotonic trend with increasing chemical complexity.

Since κ_L_ is governed by both phonon group velocity and relaxation time, we analyzed these properties to understand the observed trend. Figure 4F shows that NbFeSb overall exhibits much higher group velocities compared to the higher-entropy compounds, especially for optical phonons. In the low-frequency region (0 to 1.0 THz) where acoustic modes dominate, however, the group velocities are similar across the high-entropy systems. Figure 4G presents the phonon relaxation times, which decrease monotonically with the number of constituent elements among the high-entropy compounds. However, there is no consistent trend in relaxation times when comparing NbFeSb to the high-entropy materials. Notably, in the low–optical frequency region (1 to 4 THz), the high-entropy compounds even exhibit longer relaxation times than NbFeSb, which contrasts with the common belief that high entropy always leads to enhanced phonon scatterings. This behavior indicates that alloy scattering in these systems is not governed solely by mass disorder or the number of constituent elements. Instead, it is also strongly influenced by the specific elemental combinations, which modify local bonding environments and force-constant distributions. Our findings suggest that the phonon scattering rate is not the primary factor responsible for the reduced lattice thermal conductivity in the high-entropy compounds. Consequently, neither the phonon relaxation times nor the corresponding mean free paths exhibit a simple monotonic dependence on the degree of compositional complexity among the high-entropy compounds. Consistent with our previous findings (*20*), we find that the reduction in the diagonal component of lattice thermal conductivity from NbFeSb to the high-entropy compounds is primarily driven by decreased phonon group velocities, while the more subtle reductions among the high-entropy compounds arise from enhanced anharmonic scattering that shortens relaxation times. As a result, the phonon mean free paths calculated from these parameters, shown in Fig. 4H, correlate well with the observed trends in κ_L_.

**Fig. 5.**
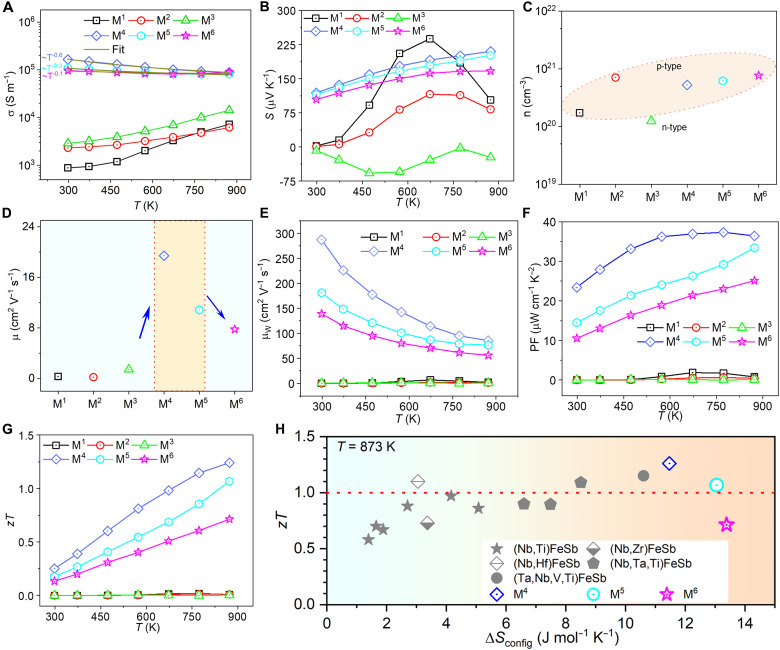
Electrical transport and TE figure of merit of M^x^FeSb. Temperature dependence of (**A**) σ and (**B**) *S* for M^x^FeSb. (**C**) *n* and (**D**) μ of M^x^FeSb alloys at room temperature. Temperature dependence of (**E**) weighted mobility, (**F**) PF, and (**G**) *zT* of M^x^FeSb. (**H**) *zT* is plotted as a function of Δ*S*_config_ for the M^4^, M^5^, and M^6^ samples, and compared with the p-type state-of-the-art (Nb, Ta) FeSb-based hH TE materials (*28*, *31*, *35*, *36*).

Entropy engineering in TE materials can enhance μ, but excessive Δ*S*_config_ often introduces strong disorder scattering that reduces μ, thereby limiting the electrical conductivity (σ) in the high-entropy alloys. The temperature dependence of σ for M^x^FeSb alloys is plotted in Fig. 5A. The σ of high-entropy M^x^FeSb (M^4^FeSb-M^6^FeSb) at room temperature increases by nearly two orders of magnitude from the low-entropy M^1^FeSb. Furthermore, the temperature dependence of σ reveals a gradual evolution in the electrical transport behavior from typical semiconductors to metal-like characteristics. Specifically, M^1^FeSb, M^2^FeSb, and M^3^FeSb show typical semiconductor behavior, while M^4^FeSb, M^5^FeSb, and M^6^FeSb demonstrate metal-like behavior. To elucidate the prevailing charge transport mechanism in the high-entropy M^x^FeSb, we used a semiquantitative power-law analysis, σ ~*T*^−α^ (*44*). According to the power-law relationship, acoustic phonon scattering is the dominant carrier scattering, with σ following ~*T*^−1.5^ dependence. This is the primary carrier scattering mechanism of low-entropy hH TE materials (*28*). However, in the case of high-entropy materials, the atomic-level disorder introduces local potential fluctuations, leading to additional scattering, disordered or alloy scattering, which is typically characterized by σ ~*T*^−0.5^ behavior. In our studied system, M^4^FeSb follows σ ~*T*^−0.6^, while M^5^FeSb and M^6^FeSb exhibit σ ~*T*^−0.3^ and σ ~*T*^−0.1^ dependence, respectively. The reduction in the exponent α, with increasing Δ*S*_config_, suggests that disorder or alloying scattering dominates the electrical transport properties. The observed α = 0.1 for M^6^FeSb is likely due to mixed scattering of alloying and the phase boundary. Although M^4^FeSb exhibits enhanced electrical conductivity, further increase in Δ*S*_config_ intensifies disorder scattering, which can lead to a reduction in μ.

The Seebeck coefficient (*S*) remains positive over the entire measured temperature regime for all the materials except M^3^FeSb, indicating holes as the dominant charge carriers (Fig. 5B). However, for M^3^FeSb, *S* becomes negative, signifying that the primary charge carriers are electrons. For the high-entropy composition (M^4^FeSb – M^6^FeSb), *S* is substantially large and increases monotonically with temperature. The carrier concentration (*n*) of M^x^FeSb alloys is in the range of (2 to 8) × 10^20^ cm^−3^ and increases slightly with increasing Δ*S*_config_ (Fig. 5C). The carrier mobility of the sample demonstrates a nonmonotonic dependence on Δ*S*_config_ and attains a maximum for M^4^FeSb before declining upon further increase of Δ*S*_config_. This indicates that high-entropy engineering does not inherently guarantee the preservation of high μ or superior electrical transport properties; there exists a critical entropy beyond which the pronounced alloy scattering degrades the mobility. Therefore, to achieve simultaneously high σ and low κ, Δ*S*_config_ of the material must be precisely tuned to balance these competing parameters. To further elucidate the impact of high-entropy engineering on electrical transport properties, we have calculated the weighted carrier mobility (μ_w_) as a function of temperature that accounts for the electrical transport as well as the band structure (*45*). As shown in Fig. 5E, μ_w_ reaches a maximum for M^4^FeSb composition and subsequently decreases. The higher μ_w_ implies a high *PF* of the material (Fig. 5F), and the average *PF* is maximum for M^4^FeSb.

Simultaneous optimization of κ_L_ and μ via high-entropy engineering led to high *zT* in M^x^FeSb material (Fig. 5G). The material, M^4^FeSb with Δ*S*_config_ ~1.4 R, demonstrates a high *zT* of about 1.25 at 873 K, while M^5^FeSb with a substantial Δ*S*_config_ ~1.6 R shows a *zT* of about 1 at 873 K, which are comparable to the low-entropy p-type hH materials (Fig. 5H) (*28*, *31*, *35*, *36*). It is evident from Fig. 5H that high-entropy compositions, M^x^FeSb, demonstrate better TE performance compared to low-entropy MFeSb materials, which is primarily due to the reduced κ_L_. Moreover, high-entropy compositions simultaneously show high *zT* and low κ. These results indicate that the incorporation of high-entropy engineering principles with an understanding of appropriate Δ*S*_config_ in conjunction with the conventional approaches provides a new direction to obtain exceptionally large *zT*.

**Fig. 6.**
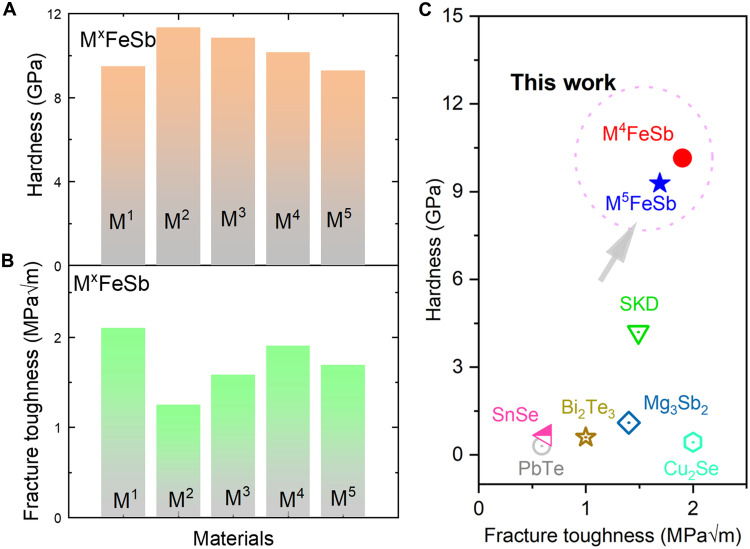
Mechanical properties of M^x^FeSb. (**A**) Vickers hardness and the (**B**) fracture toughness of M^x^FeSb alloys. (**C**) Vicker hardness and fracture toughness of M^x^FeSb with various state-of-the-art TE materials (*2*), such as PbTe-based (*49*), SnSe-based (*50*), Cu_2_Se-based (*51*), Skutterudite (SKD)–based (*52**,* *53*), Mg_3_Sb_2_-based (*53*), Bi_2_Te_3_-based (*54*), and low-entropy hH-based (*48*).

Next, we studied the mechanical properties to ensure that high-entropy design does not lead to a deterioration of mechanical stability. To fabricate sustainable TE devices that can withstand in-service loading, adequate hardness and fracture toughness of the materials are required (*2*, *46*). We measured the Vickers hardness and approximated the fracture toughness of single-phase M^x^FeSb materials (Fig. 6, A and B). The increased values of hardness in high-entropy compositions compared to the NbFeSb composition, consistent with these samples having a smaller grain size than the NbFeSb sample, indicate improved resistance to mechanical deformation during operation. The incorporation of Ta results in the greatest hardness and is consistent with TaFeSb having a greater reported hardness than NbFeSb (*47*). Herein, with 50% Ta substitution in place of Nb in M^2^FeSb yields the maximum hardness, and after that, the hardness value starts to decrease with the addition of Ti, V, and Hf in high-entropy compositions (Fig. 6A). The approximated fracture toughness values are within the 1.3- to 2.3-MPa√m range for conventional hH alloys (*46*), and despite a strength (hardness)–ductility (fracture toughness) trade-off, M^4^FeSb and M^5^FeSb show reasonably high hardness and fracture toughness, which is higher compared to reported low-entropy hH TE materials (*48*). The mechanical properties of the high-entropy M^x^FeSb are notably higher than various classes of state-of-the-art TE materials (Fig. 6C) (*2*, *48*–*54*). The hardness values of M^x^FeSb are within the range of other hH alloys (*46*) and exceed those of the other classes of TE materials as the crystal structure does not have soft directions of layered TE materials (e.g., SnSe, Bi_2_Te_3_, and Mg_3_Sb_2_), sublattice disorder (Cu_2_Se), weak bond strength (e.g., PbTe), or large structural voids in the crystal structure (e.g., Skutterudites).

## DISCUSSION

In summary, we have successfully synthesized entropy-engineered hH TE materials, M^x^FeSb, which demonstrate that the lattice thermal conductivity reduces asymptotically with increasing configurational entropy, and beyond a critical entropy level, further reduction in thermal conductivity becomes negligible. While the carrier concentration of the M^x^FeSb is relatively insensitive, the carrier mobility is strongly influenced by the high-entropy design that induces high electrical conductivity in M^x^FeSb. However, the carrier mobility reduces at higher entropy due to intensified disorder scattering. Through simultaneous optimization of configurational entropy, carrier mobility, and lattice thermal conductivity, M^4^FeSb demonstrates a high TE figure of merit. In addition, the high-entropy compositions show superior mechanical properties in terms of Vickers’ hardness and fracture toughness. Our study suggests that the incorporation of high-entropy engineering with appropriate configurational entropy, in conjunction with conventional approaches, can result in a higher figure of merit for any class of TE materials.

## MATERIALS AND METHODS

### Material and synthesis

The entropy-engineered M^x^FeSb hH alloys such as NbFeSb (denoted as M^1^FeSb) Nb_0.5_Ta_0.5_FeSb, (denoted as M^2^FeSb) Nb_0.33_Ta_0.33_Ti_0.33_FeSb (denoted as M^3^FeSb), Nb_0.25_Ta_0.25_Ti_0.25_V_0.25_FeSb (denoted as M^4^FeSb), Nb_0.225_Ta_0.225_Ti_0.225_V_0.225_Hf_0.1_FeSb (denoted as M^5^FeSb), and Nb_0.2_Ta_0.2_Ti_0.2_V_0.2_Hf_0.2_FeSb (denoted as M^6^FeSb) were synthesized using high-energy ball milling followed by spark-plasma sintering (SPS) process. The high-purity constituted elements Nb (99.9%, foil), Ta (99.99%, foil), Ti (99.9%, wire), V (99.99%, piece), Hf (99.9%, piece), Fe (99.995%, piece), and Sb (99.999%, shots) were weighed according to the required molar ratio and loaded into a stainless steel jar inside a glove box to maintain the inert Argon atmosphere. The raw elements were ball-milled using an SPEX mixer/mill (model 8000D, SPEX SamplePrep, Metuchen, NJ, USA) for 20 hours to ensure homogeneity. The powder was loosened after 12 hours of mechanical alloying and subsequently, every 2 hours. The alloyed powders were consolidated via SPS (Dr. Sinter-625 V, Fuji, Japan) at 1098 K under a pressure of ~80 MPa for 2 min. All the SPS samples have a relative density of 99% of the theoretical value. The obtained SPS pellets were cut into rectangular bars (~2 mm by 2 mm by 12 mm) for electrical transport measurements.

### Crystal structure and microstructure characterization

XRD patterns of the SPS samples were collected at room temperature on a polished surface by an x-ray diffractometer (PANalytical Empyrean) using Cu-K_α_ radiation over a 2θ range of 20° to 80°. The microstructures were characterized by FESEM (FEI Verios G4), EDS (Oxford Aztec), and EBSD (FEI Apero S). The grain size and grain boundary distribution were analyzed using Oxford Aztec Crystal software.

### TE properties measurement

The temperature dependence of σ and *S* were measured simultaneously using a commercial ZEM-3 system (ULVAC-RIKO, Japan) under a He atmosphere. κ was calculated by $\kappa =D\rho_{d}C_{p}$, where *D*, ρ_d_, and *C*_p_ represent thermal diffusivity, density, and specific heat capacity of the sample, respectively. Thermal diffusivity was measured by a laser flash system (LFA-467 HT HyperFlash, Germany). *C*_p_ was measured using differential scanning calorimetry (Netzsch, Germany). The density of the sample was measured by using Archimedes’ principle. The uncertainties in the measurement of σ, κ, *S*, and *zT* were estimated to be ±5, ±2, ±5, and ±7%, respectively. $\kappa_{e}$ is proportional to σ following the Weidman-Franz law, $\kappa_{e}=L\sigma T$, where *L* is the Lorentz number, and *T* is the absolute temperature. *L* was calculated as a function of temperature based on the measured *S* using $L=1.5+\text{exp}\left(-\frac{|S|}{116}\right)$, (where *L* is in 10^−8^ WΩK^−2^ and *S* in μV/K) (*38*). $\kappa_{l}$ was calculated by subtracting $\kappa_{e}$ from κ. The Hall coefficient $\left(R_{H}\right)$ was measured using a Hall effect measurement set-up (8400 Series HMS, LakeShore). The carrier concentration $\left(n_{H}\right)$ and hole mobility $\left(\mu_{H}\right)$ were calculated using,$n_{H}=1/\left(eR_{H}\right)$ and $\mu_{H}=R_{H}\sigma$, where *e* is the electron’s charge. The weighted mobility $\left(\mu_{W}\right)$ is calculated from the Seebeck coefficient and electrical resistivity (ρ) as (*45*)

$$\mu_{W}=\frac{331}{\rho }{\left(\frac{T}{300}\right)}^{-3/2}\left\{\frac{\text{exp}\left[\frac{|S|}{k_{B}/e}-2\right]}{1+\text{exp}\left[-5\left(\frac{|S|}{k_{B}/e}-1\right)\right]}+\frac{\frac{3}{\pi^{2}}\left[\frac{|S|}{k_{B}/e}\right]}{1+\text{exp}\left[5\left(\frac{|S|}{k_{B}/e}-1\right)\right]}\right\}$$ (1)

where $\mu_{W}$ is in cm^2^ V^−1^ s^−1^, ρ is in mΩ·cm, T is in K, and $k_{B}/e$ ~ 86.3 μV K^−1^.

### Sound velocity measurement

The longitudinal (ʋ_L_) and transverse (ʋ_t_) sound velocity of M^x^FeSb were measured by a pulse-echo method using an ultrasonic instrument. The measurement process involved one transducer functioning as a transmitter, generating the ultrasound signal, while another acted as a receiver. Both transducers shared identical dimensions and frequency ranges. Using a Hamming window with seven-cycle pulses for time propagation measurement enabled the acquisition of sound velocity data. For the generation of longitudinal and transverse ultrasonic bulk waves, Steminc transducers SMD10T2R111 (1 MHz) and SM4113 (525 kHz) were used, respectively. Parylene-C coating (≃6 μm) was applied to the transducers to prevent unwanted electrical shorts during measurements. To produce and capture ultrasound signals, the two transducers were positioned at an adequate distance within a water medium, aiming to prevent any undesired ringing artifacts originating from the transducers. The average sound velocity (*v_s_*) was calculated using the following equation

$$v_{s}={\left[\frac{1}{3}\left(\frac{1}{v_{l}^{3}}+\frac{2}{v_{T}^{3}}\right)\right]}^{-1/3}$$ (2)

### Mechanical properties measurement

Fine polished samples were used to measure the mechanical properties. The Vickers hardness and fracture toughness were measured using the LM110AT microhardness testing system (LECO Corporation) with a dwell time of 10 s, and 15 measurements were made for each composition. The crack lengths emanating from the Vickers indentations were measured with a VHX-2000 optical microscope (Keyence Corporation), and fracture toughness was approximated as $K_{\mathrm{IC}}=0.0319\frac{P}{a\sqrt{l}}$, where *P* is the indentation load, *a* is the half-diagonal indentation length, and *l* is the Palmqvist crack length (*55*).

### Theoretical calculations details

We performed first-principles simulations using the Vienna ab initio simulation package (VASP) (*56*–*59*) within the framework of density functional theory (DFT), using the projector-augmented wave (PAW) (*60*) method and the revised Perdew-Burke-Ernzerhof for solids (PBEsol) (*61*) to calculate the total energies and forces of NbFeSb, (NbTa)FeSb, (NbTaTi)FeSb, and (NbTaTiV)FeSb. The structures of the high-entropy compounds were generated using the icet package (*62*), with Nb sites randomly occupied by additional elements while maintaining overall stoichiometry.

For NbFeSb, structural relaxation was performed using VASP. A kinetic energy cutoff of 350 eV and a Γ-centered Monkhorst-Pack k-point mesh of 20 × 20 × 20 were used for the three-atom primitive cell. Convergence criteria for total energy and force were set to 10^−6^ eV and 10^−3^ eV/Å, respectively. For the high-entropy systems, which comprise 324 atoms per conventional cell, structural relaxation was performed via molecular dynamics (MD) simulations using on-the-fly machine learning (ML) (*63*–*65*) force fields implemented in VASP. The MD simulations were conducted at 300 K with a 2-fs time step for 4000 steps (8 ps total).

Harmonic and anharmonic interatomic force constants (IFCs) for all four compounds were obtained using Phonopy (*66*) and thirdorder.py from ShengBTE package (*67*). For NbFeSb, both harmonic and third-order anharmonic IFCs were computed using a 4 × 4 × 4 supercell, with a cutoff radius of 0.8 nm applied for the anharmonic case. In contrast, for the high-entropy compounds, 1 × 1 × 1 supercells were used for both harmonic and anharmonic IFCs, with a smaller cutoff of 0.3 nm for the latter. Lattice thermal conductivities were computed using the ShengBTE package (*67*) with a broadening parameter (scalebroad) of 0.1 applied to all calculations, where the Boltzmann transport equation (BTE) was primarily solved within the relaxation time approximation (RTA) by consideration of only three-phonon scattering processes. To validate this approach, additional calculations were performed using the self-consistent BTE solver, yielding lattice thermal conductivity values very close to those obtained within the RTA. For the conductivity calculations, a 12 × 12 × 12 q-point mesh and 4 × 4 × 4 supercell were used for NbFeSb, whereas a 3 × 3 × 3 q-point mesh and 1 × 1 × 1 supercell dimensions were applied for the high-entropy compounds. In addition, the lattice thermal conductivity was decomposed into diagonal and off-diagonal contributions based on the unified theory of heat transfer (*41*).
