# Supplementary material for: Understanding the role of entropy in designing high-performance thermoelectrics
Source: Sci Adv. 2026 Jul 3;12(27):eaed6943. doi: 10.1126/sciadv.aed6943 (PMC13330816; doi:10.1126/sciadv.aed6943)
Supplement: Supplementary file 1 — Supplementary Text S1 and S2 Figs. S1 to S8 [file sciadv.aed6943_sm.pdf]

Science Advances
AAAS

Supplementary Materials for

Understanding the role of entropy in designing high-performance thermoelectrics

Subrata Ghosh *et al.*

Corresponding author: Subrata Ghosh, smg7204@psu.edu; Yi Xia, yxia@pdx.edu; Bed Poudel, bup346@psu.edu

Sci. Adv. **12**, eaed6943 (2026)
DOI: 10.1126/sciadv.aed6943

This PDF file includes:

Supplementary Text S1 and S2
Figs. S1 to S8

Note S1: Calculation of configuration entropy (ΔS_{config}).

We have calculated the configurational entropy (ΔS_{config}) in a single Wyckoff site of the high entropy engineered materials, M^xFeSb, using the following equation *(25)*,

$$\Delta S_{config} = -R \sum_{i=1}^{n} x_i ln x_i \tag{S1}$$

where n is the number of elements with the mole fraction x_i. Here R is the universal gas constant, 8.314 J K^{-1} mol^{-1}.

Note S2: Calculation of minimum thermal conductivity using the Cahill model.

We have calculated the minimum thermal conductivity (κ_{min}) of NbFeSb half-Heusler alloy, using the Cahill model *(33)*,

$$\kappa_{min} = \left(\frac{\pi}{6}\right)^{1/3} k_B n^{2/3} \sum_i v_i \left(\frac{T}{\Theta_i}\right)^2 \int_0^{\Theta_i/T} \frac{x^3 e^x}{(e^x-1)^2} dx \tag{S2}$$

Here, the summation is over two transverse and one longitudinal effective acoustic polarization with a sound velocity of v_i, which essentially accounts for all the phonon modes. Θ_i is the cutoff frequency for each polarization, and is defined as, $\Theta_i = v_i \left(\frac{\hbar}{k_B}\right)(6\pi^2 n)^{\frac{1}{3}}$. n is the number density of atoms.

In the high-temperature limit, where all vibrational modes are thermally excited, the predicted minimum thermal conductivity can be estimated as *(34)*.

$$\kappa_{min} = 0.4\, k_B n^{2/3}(v_l + 2v_t) \tag{S3}$$

Using the measured density and longitudinal and transverse sound velocities, the minimum thermal conductivity is calculated to be ~ 0.92 W m^{-1} K^{-1}, which is very similar to the value estimated for other MFeSb half-Heusler materials *(31)*.

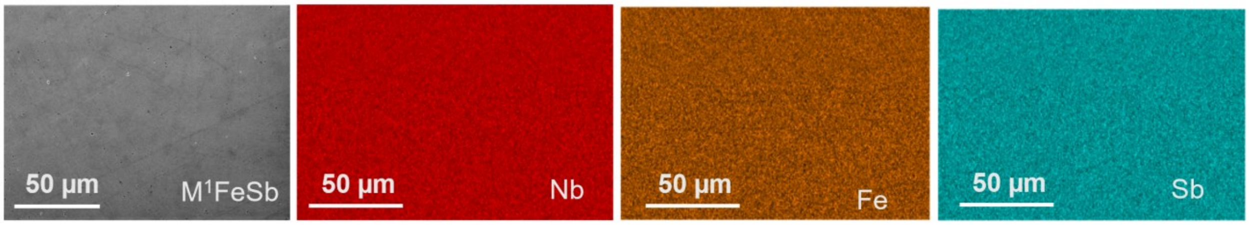

Fig. S1.

Elemental maps of M^1FeSb. FESEM image and the EDS mapping of M^1FeSb showing the homogeneous distribution of constituent elements within a micrometer length scale.

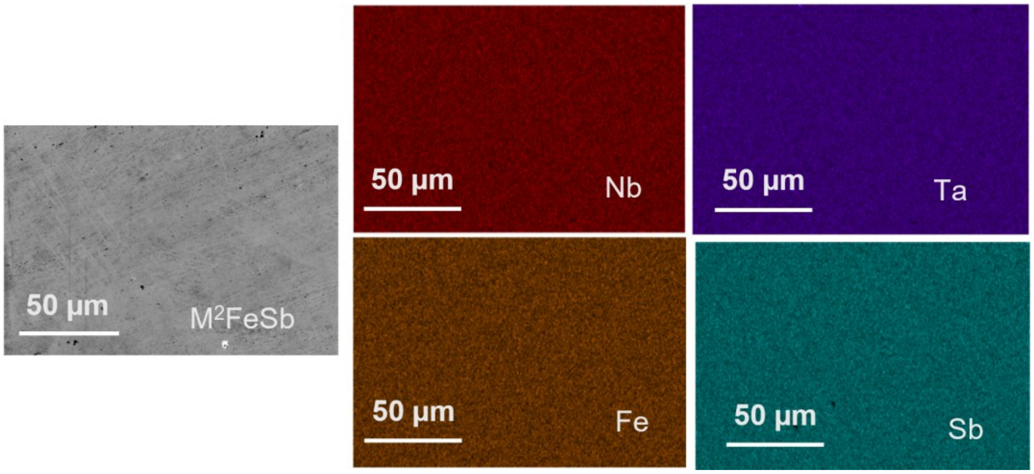

Fig. S2.

Elemental maps of M²FeSb. FESEM image and the EDS mapping of M²FeSb show the homogeneous distribution of constituent elements within a micrometer length scale.

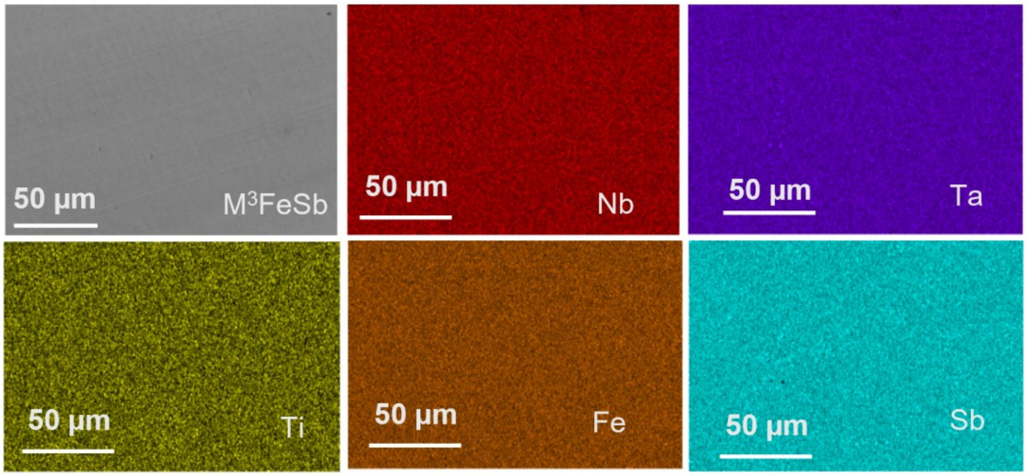

Fig. S3.

Elemental maps of M^3FeSb. FESEM image and the EDS mapping of M^3FeSb showing the homogeneous distribution of constituent elements within a micrometer length scale.

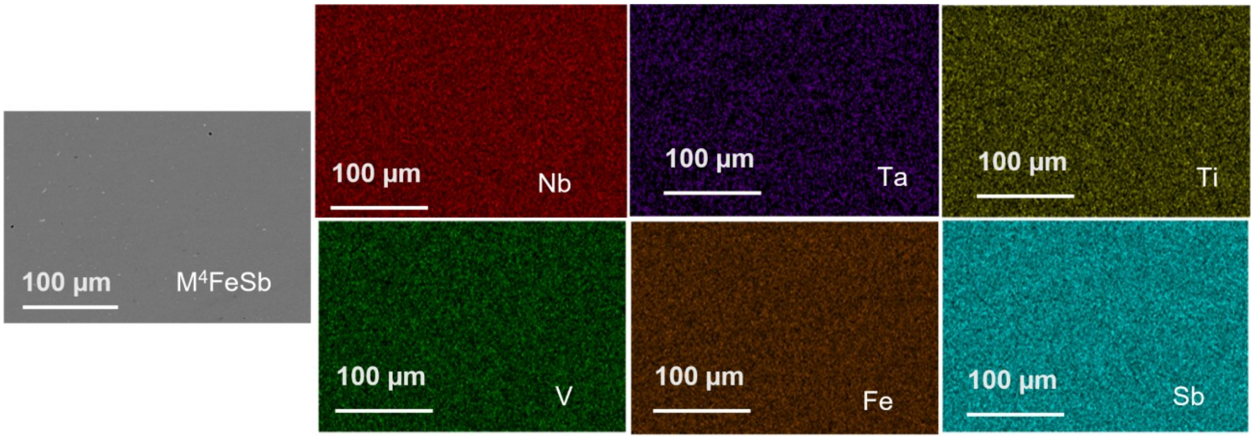

Fig. S4.

Elemental maps of M^{4}FeSb. FESEM image and the EDS mapping of M^{4}FeSb showing the homogeneous distribution of constituent elements within a micrometer length scale.

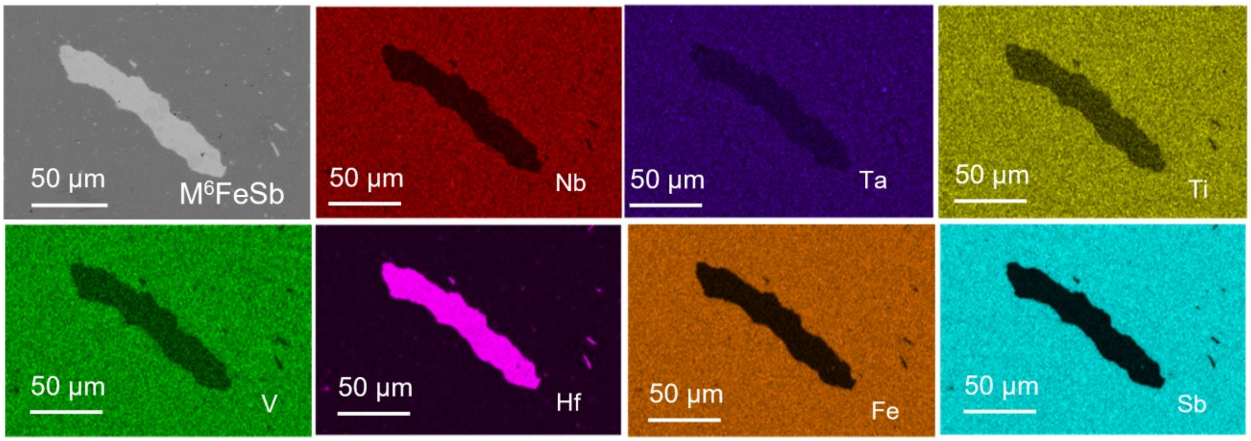

Fig. S5.

Elemental maps of M^6FeSb. FESEM image and the EDS mapping of M^6FeSb showing the Hf-enriched micro-precipitates, and it acts as a secondary phase in the matrix.

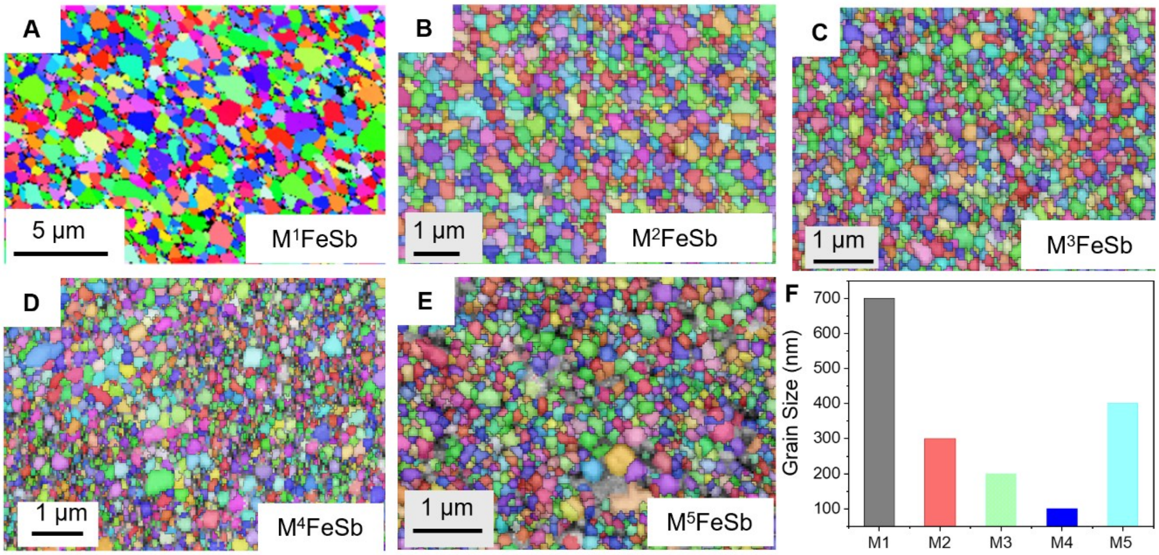

Fig. S6.

EBSD Characterization of M^{x}FeSb. EBSD analysis of (**A**) M^{1}FeSb, (**B**) M^{2}FeSb, (**C**) M^{3}FeSb, (D) M^{4}FeSb, and (E) M^{5}FeSb showing the grain boundaries and random grain orientation. (**F**) Comparison of the average grain size for M^{x}FeSb alloys.

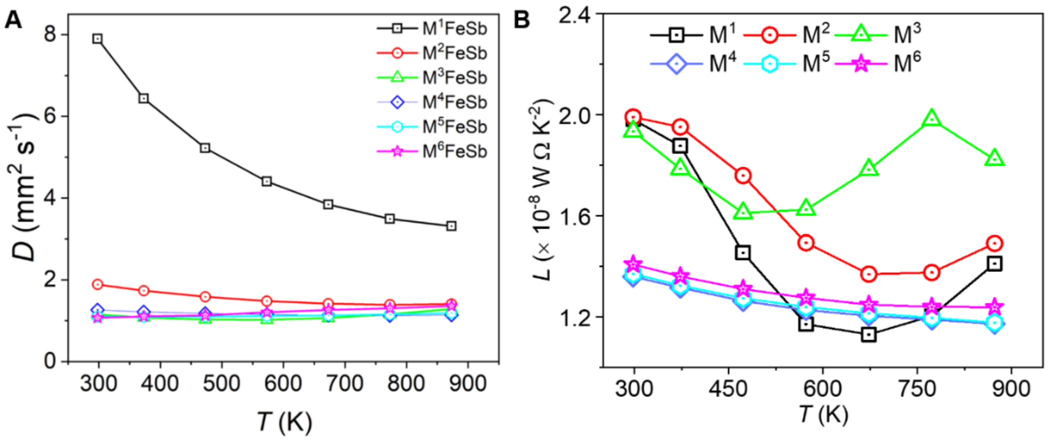

Fig. S7.

Thermal diffusivity and calculated Lorentz number of M*x*FeSb. Temperature dependence of (**A**) thermal diffusivity, and (**B**) the calculated Lorentz number of M*x*FeSb alloys.

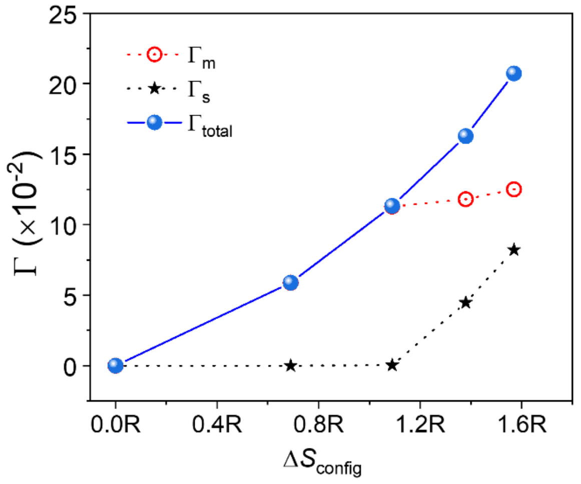

Fig. S8.

Mass and strain-field fluctuation parameters of M^{x}FeSb. The calculated total disorder parameter, Γ_{tot}, associated with mass (Γ_M) and strain-field fluctuations (Γ_S), as a function of configurational entropy (ΔS_{config}) for M^{x}FeSb
